# IxPopDyMod: an R package to write, run, and analyze tick population and infection dynamics models

**DOI:** 10.1186/s13071-024-06171-2

**Published:** 2024-02-26

**Authors:** Myles Stokowski, David Allen

**Affiliations:** Department of Biology, Middlebury College, Middlebury, 05753 VT USA

**Keywords:** Tick population dynamics, R package, IxPopDyMod, *Ixodes scapularis*, *Dermacentor albipictus*, *Borrelia burgdorferi*

## Abstract

**Abstract:**

Given the increasing prevalence of tick-borne diseases, such as Lyme disease, modeling the population and infection dynamics of tick vectors is an important public health tool. These models have applications for testing the effects of control methods or climate change on tick populations. There is an established history of tick population models, but code for them is rarely shared, especially not in a convenient format for others to modify and use. We present an R package, called IxPopDyMod, intended to function as a flexible and consistent framework for reproducible Ixodidae (hard-bodied ticks) population dynamics models. Here we focus on two key parts of the package: a function to create valid model configurations and a function to run a configured model and return the daily population over time. We provide three examples in appendices: one reproducing an existing *Ixodes scapularis* population model, one providing a novel *Dermacentor albipictus* model, and one showing *Borrelia burgdorferi* infection in ticks. Together these examples show the flexibility of the package to model scenarios of interest to tick researches.

**Graphical Abstract:**

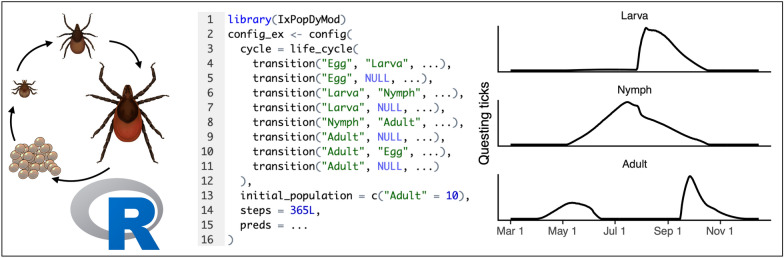

**Supplementary Information:**

The online version contains supplementary material available at 10.1186/s13071-024-06171-2.

## Background

Ticks are obligate, blood-feeding parasites and vectors of important wildlife, livestock, and human pathogens [1]. It is important to understand tick population and infection dynamics given their impacts on public health [2]. This is hard because determining true tick population sizes is difficult [3] and many tick life stages and processes are difficult to directly observe or measure.

Models of tick populations offer important tools to understand population dynamics. They can be used to predict how tick populations will respond to climate change [4–6], to test potential tick and tick-borne disease control strategies [7, 8], or to interrogate theories of tick-borne disease transmission that are hard to address experimentally [9–11]. There is a long history of modeling tick populations [7, 12, 13]. These models can take a number of different forms including ordinary differential equations [14, 15], difference equations [16], Leslie models [17, 18], and agent-based models [19]. As with other models there is a trade-off between model simplicity and ability to arrive at analytic results versus model complexity and the need for simulation.

Unfortunately for some of these tick population models, the computer code to run the models is not included. This makes it hard for others to modify parts of the analysis, assess sensitivity of parameters, or provide full transparency of model assumptions [20]. Ultimately if model details are unavailable this limits the trust in using models to inform policies and decisions [21]. We present IxPopDyMod (= Ixodidae Population Dynamics Model), a framework for specifying and running mechanistic models of Ixodidae population and infection dynamics. IxPopDyMod is structured as an R package which is available on CRAN [22]. In this way, a model can be fully specified with a relatively simple R file. From there the model can be re-run or modified by other users with small changes to that R file. The package makes it easier for non-experts in R to write tick populations models, share their code, and modify models created by others.

IxPopDyMod supports classic matrix models, where the vector of current life stage population sizes is multiplied by a matrix of transition probabilities, giving the vector of population sizes at the subsequent time step. However, this approach alone does not accurately represent Ixodidae tick life cycles. In many cases, transitions between tick life stages occur over a multi-day period. To accommodate this, the modeling framework supports duration-based transitions, where ticks undergo a developmental period lasting more than one day. Both the duration of these transitions and the probability of the matrix model-style transitions are determined by user-specified functions which can depend on predictors, such as host population size or temperature.

## Model configuration

**Fig. 1:**
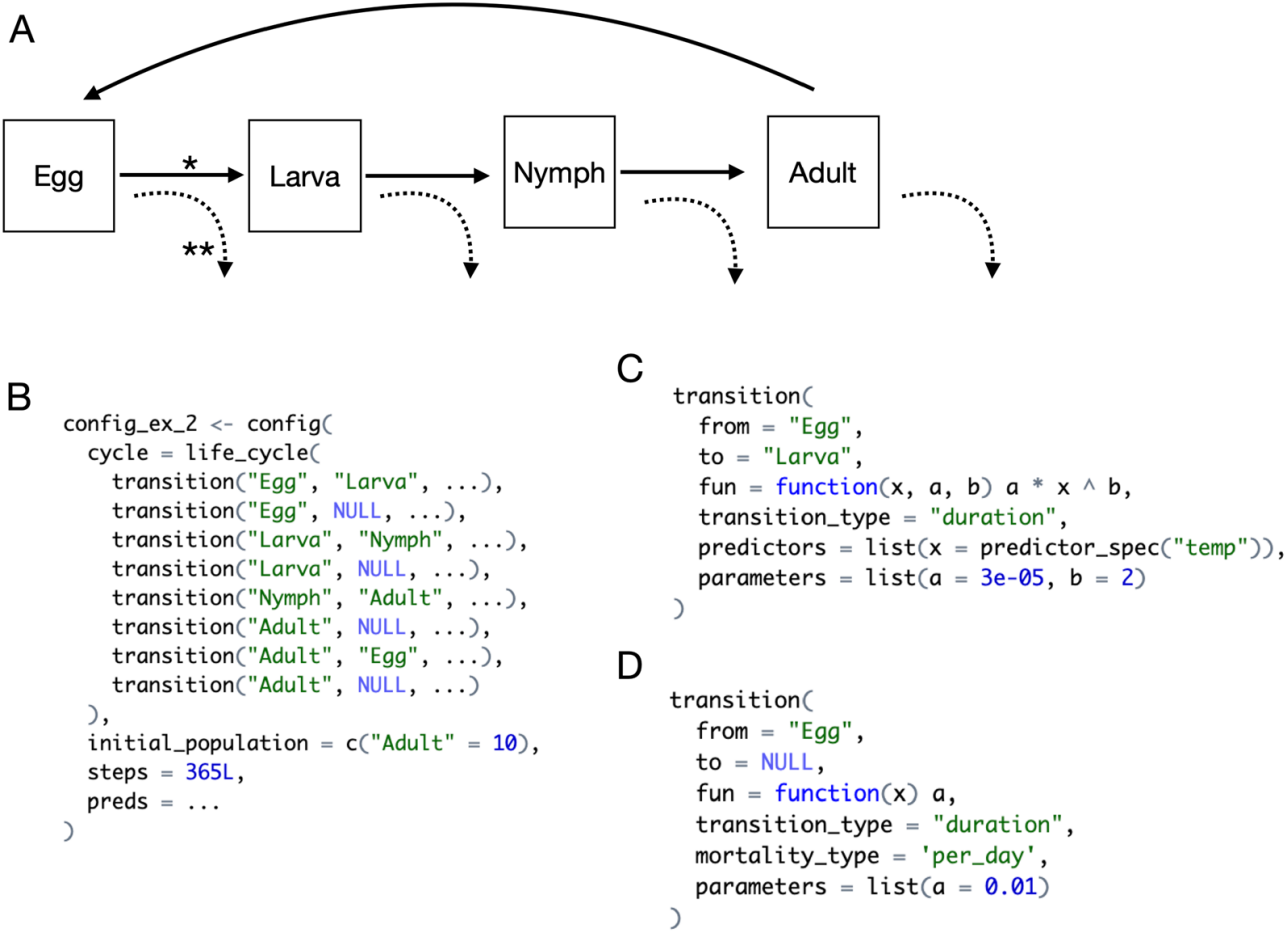
Overview of specifying a config. **A** Life cycle flow diagram for a modeled tick population. Solid arrows represent interstadial development between life stages or egg laying. The dashed downward-curving arrows represent mortality. **B** The config code for this tick life cycle. Each arrow in the diagram is a transition object in the life_cycle. In this case the model would start with 10 adults and run for 365 days. For brevity some code is omitted with “...”. **C** The full code for the first transition marked with an * in 1A. This indicates that the transition from egg to larva is a duration transition whose rate is an exponential function of temperature. This function has parameter values provided for a and b. We specify that the formal argument x should get values from predictor data labeled as temp—this assumes that we are working with a config containing such predictor data (preds argument). **D** The full code for the second transition marked with ** in 1A. This indicates that every day eggs are developing into larvae they have a 1% mortality rate

Modeling tick populations using IxPopDyMod requires a single input object called a config, which is sufficient to specify a model configuration with reproducible results. An example config is shown in Fig. 1. Others are shown in Additional file 1: Appendices A–C and included in the package [22]. Users may modify these or build new ones from scratch.

Constructing a config runs a suite of validation checks to guide users creating their own model configurations and catch errors before runtime. The separation of the inputs that configure the model from the code that validates and runs the model makes IxPopDyMod a flexible and convenient framework for modeling various tick populations, without modifying underlying code.

**Fig. 2:**
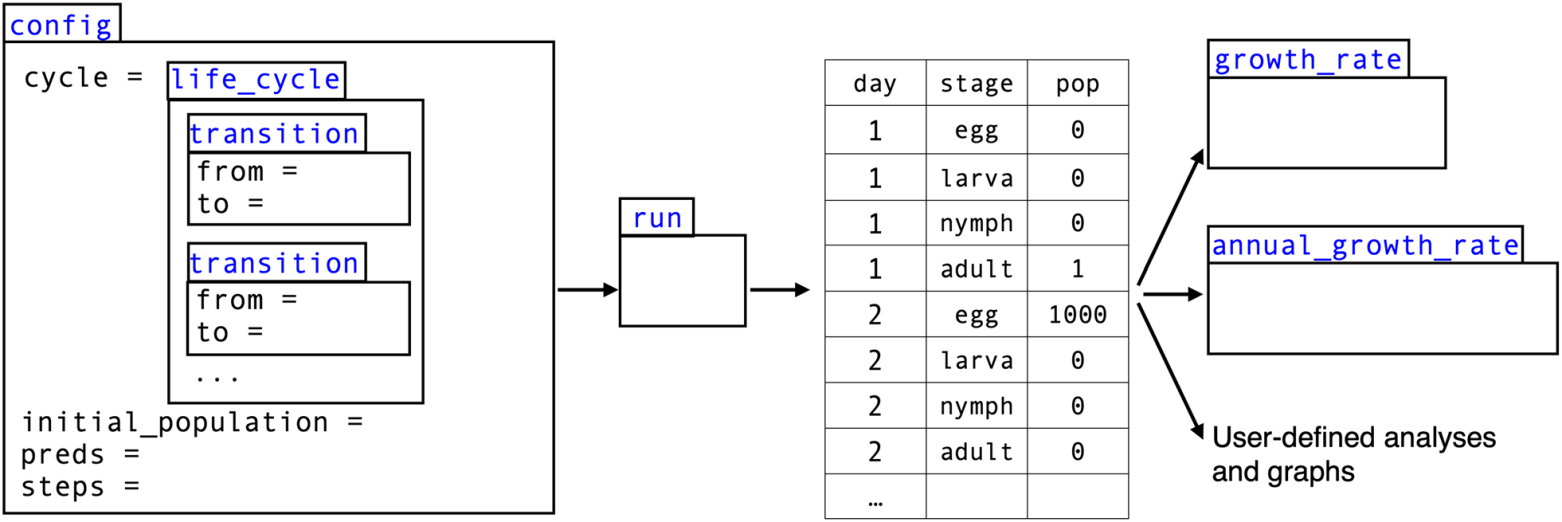
A conceptual scheme of how the six core commands which a user will use relate. Commands are represented by tabbed rectangles with their name in blue. Some arguments of commands are listed inside the rectangle. The output of run is a data frame. Example user-defined analyses and graphs can be seen in Additional file 1: Appendices A–C

The required components of a config are: a life_cycle object representing transitions between tick life stages and the initial population size of each tick life stage (Figs. 1B and  2). Users may optionally specify input predictor data for transitions to depend on, for example tick host density or weather data. These data can be variable or constant throughout the modeling period. Additional configuration options, with default values, are the number of time steps to run the model, the maximum number of time steps that ticks can remain in a duration-based transition, and whether to print verbose logs (Table 1).

**Table 1:** Six core commands in the package which a user will use when specifying, running, and analyzing a model

Command	Inputs	Description
config	cycle, initial_population, steps, preds, max_duration	This command makes a configuration object which is used to run the model. It is a convenient way to make the model shareable. These configs can be modified to run the model under different climate, host community scenarios, or different parameter values
life_cycle	Multiple transitions	The life cycle object is required for the config. It specifies the tick life cycle as a series of transitions between life stages
transition	from, to, fun, transition_type, mortality_type, parameters, predictors	A transition is a single step in the tick life cycle, from one life stage to the next. Each transition is either a probability of a tick going from one life stage to the next (e.g. probability a questing tick finds a host) or the duration of time that transition takes (e.g. how long it takes a developing egg to hatch). These probabilities or durations are given as a function (can be constant) which could have parameters and/or take predictors
run	config	This command runs the model from a config object
growth_rate	run output	This command calculates the daily growth rate of a population from the output of run
annual_growth_rate	run output	This command calculates the annual growth rate of a population from the output of run

The life_cycle and predictors are the main components that allow IxPopDyMod to be flexibly configured. Here, we detail their structure, how they are interpreted when the model is run, and the variability in Ixodidae biology that these configurations capture.

### Life cycles

IxPopDyMod life_cycles are hierarchical data structures that represent tick life cycles. A life_cycle is composed of a list of transition objects, each of which includes the names of the origin and destination life stages (Figs. 1B and 2). The package will accept any life-stage names the user provides, but this will generally be egg, larva, nymph, and adult (Fig. 1A) or finer gradations of these stages (e.g. hardening larva, questing larva, feeding larva, etc., Additional file 1: Appendix A). Each transition includes a fun argument, which must be an R function that is evaluated to a numeric vector as the model runs. Transition functions can be drawn from a bank provided in the package or a user-defined custom function (for examples see Fig. 1C,D or Appendix C). A transition level configuration option allows toggling between interpreting this transition value as either a daily probability of transitioning between life stages or as a duration until ticks emerge into the next life stage. Optionally, transition functions can take parameters, specified using a parameters object, and a list of predictors_spec objects, which determine how predictor data should be used in evaluating a transition. The total population of a set of tick life stages may also be used as predictor data, as configured via an argument in predictors_spec(). This allows for transition probabilities to be density dependent. For example, in Appendix A on-host mortality is density-dependent based on the number of ticks attached and feeding on a host.

Transitions may also be configured to represent the mortality probability from a given life stage rather than a transition between two life stages (Fig. 1D). When configuring mortality probabilities, transition functions can be used to calculate either the daily mortality probability or the probability over the entire course of a duration-based transition. A full list of transition arguments (some optional) is given in Table 1.

### Predictors

Tick population dynamics are affected by environmental conditions [23] and the density of host species [10]. IxPopDyMod allows specifying these types of predictors as a model input used in evaluating transitions. By modifying input predictor data, the model could be applied to compare tick dynamics in different climate scenarios, to simulate tick populations across space using local weather data (see Additional file 1: Appendix A), or to investigate the effects of different host densities (see Additional file 1: Appendices B, C). Users can specify any predictor they want to include in their model, for example daily average temperature (Additional file 1: Appendix A) or snow cover (Additional file 1: Appendix B). Other weather variables that influence tick survival or behavior, such as vapor pressure deficit, could be incorporated. Users can find these weather values directly from weather stations (Additional file 1: Appendix B) or from model interpolated values (e.g. PRISM [24]).

Predictors are structured as a data frame where each row stores a predictor name and value that can apply to either the entire modeling period or a specific day. The predictors object is provided to enforce the correct structure of the input data.

### Multiple host species

Many Ixodidae species feed on multiple host species. For such ticks, different host species often result in differential probability of feeding success or transmission of a tick-borne pathogen [25, 26]. IxPopDyMod supports this behavior through a combination of model configuration options at the predictors and transition levels.

In the table of predictors data, an optional column allows specifying subcategories of predictors—for example, a predictor called host_density could have subcategories for mouse and deer. A corresponding transition with a predictor_spec that indicates using host_density data would then receive a named vector, with names drawn from the pred_subcategory column in the predictors and values drawn from the value column. Users may wish to pair this with named vector parameters, for example a named vector of parameters with different values for mouse and deer. See Additional file 1: Appendices A and C for an examples of this.

## Running the model

Running the model is as simple as calling the run() method with a valid config() object. Doing so returns a data frame of daily population counts per tick life stage (Fig. 2). Model runtime depends on computer hardware and how a model is configured, notably the number of time steps. As a rough point of reference, running the ogden2005 configuration, a complex model with 12 life stages run for 3500 time steps, took 34 s on a 2020 M1 MacBook Air. Here we explain how run() interprets a model configuration and the calculations it performs throughout a model run.

IxPopDyMod is a simulation model that operates in daily time steps. On a given day, each transition is evaluated, which involves calling the transition function with any specified parameters and predictors. How the resulting transition value is used differs between probability- and duration-based transitions. For probability-based transitions the model calculates the transition probabilities between life stages. To evaluate each transition, the transition function is called with any specified parameters and predictors. The values from probability type transitions are entered into a transition matrix, where each cell indicates the probability of transitioning between a pair of life stages. Survival, or the fraction of ticks that remain in a given life stage, is calculated as $1 - (\text {sum of transition probabilities from a life stage}) - (\text {mortality from that life stage})$.

The values from duration-based transitions are used to determine the day when ticks will emerge into a subsequent stage. Duration-based transitions may either return a vector of length one, or a vector of a length specified by the max_duration setting in a config—the maximum number of days a transition may last. If the vector is of length one, the value is duplicated to length max_duration. In either case, the resulting value is interpreted as the fraction of development completed on that day—ticks emerge from a duration-based transition once the sum of the daily transition values exceeds one. In duration-based transitions, ticks either proceed to a subsequent life stage or die. Mortality values in this case control the fraction of ticks that proceed to the next stage.

To calculate the population of each life stage on the next day, the transition matrix is multiplied by the current population. Then, the number of ticks emerging into each life stage from duration-based transitions is added. Internally, run() keeps track of the number of ticks of each life stage that are undergoing duration-based transitions. Ticks undergoing delayed transitions are counted as part of the life stage they originated in until they complete development.

## Interpreting model results

The data frame returned by run() contains the number of individuals in each life stage on each day, which can be interpreted in a number of ways (Fig. 2). To calculate a general trend in the population size over time, the function annual_growth_rate() returns a scalar value representing the population’s average annual multiplicative growth rate. Other post hoc analysis could include calculating annual peak population of a specific life stage (Additional file 1: Appendix A), plotting population size over time broken out by tick life stage (Additional file 1: Appendix B), or calculating the fraction of infected versus uninfected ticks if the model includes a tick-borne pathogen (Additional file 1: Appendix C).

The model can then be run with different predictor values to see, for example, how the tick population would respond to different climate conditions (Additional file 1: Appendices A, B) or different host density (Additional file 1: Appendices B, C). Generally model parameters will be drawn from the literature, through experiments or direct observation, but users could also run the model with different parameter values to deduce parameters that are difficult to derive experimentally or in the field.

## Full model examples

In three appendices we give examples configs, runs, and analyses. In Additional file 1: Appendix A we reproduce Ogden et al. [16]’s *Ixodes scapularis* population model in our package framework. In the Appendix we show that we can reproduce the results which they present. This includes showing how the tick population responds to climates in different locations in eastern Canada. With the config provided, it is now possible for others to use or modify this population model of a tick species of key public health importance.

In Appendix B we provide a novel *Dermacentor albipictus* population model, which is parameterized from the literature. While *I. scapularis* is a three-host tick (i.e. finds a new host at each of its three life stages), *D. albipictus* finds a single host in its life and takes three blood meals from that host. This demonstrates the flexibility of the package to accommodate different tick life histories. *Dermacentor albipictus* is an important parasite of moose (*Alces americanus*) populations [27]. In the Appendix, we show how the tick population responds to different moose population densities.

In Additional file 1: Appendix C, we give an example of how infection with a tick-borne pathogen can be added to a model in our package. The example is for *Borrelia burgdorferi*, the Lyme disease agent, transmitted by a population of *I. scapularis* ticks.

## Limitations

The package provides a flexible structure to model many aspects of the diverse biology of hard ticks. We hope that its flexibility will meet the needs of most users looking to model a tick population. Still, the model has some limitations. The model assumes well-mixed, spatially homogeneous tick and host populations. It does not have a built-in structure to model landscapes made up of a fraction of different habitat types [7, 28, 29] or a spatially explicit structure [19, 30]. Still, it would be possible for the user to, for example, run the model on “patches” with and “migrate” ticks between them. For example, to model a population in forest and meadow habitats, a user could set up configs for each habitat with different parameters (e.g. different tick survival in the two habitats) and/or predictors (e.g. different host communities) and then run each config for a set period of time. Then, the user could subtract individuals of specific life stages (those that disperse, e.g. ticks on hosts) from the output in one habitat and add them to the output in the other to represent migration. This process could be repeated to see how the population in the two habitats changes over time.

Another limitation is that all hosts of a given species are assumed to be identical with the same number of ticks. However, in almost all systems parasites are highly aggregated on hosts [31]. Relatively few tick population models accommodate this fact (though see [32]). One way to deal with that in our package would be to specify two different host types of the same species, for example a “high-parasite mouse” and a “low-parasite mouse.” These two host types, though the same species, could have different tick attachment rates and tick density-dependent feeding parameters. This would not give a full distribution of different ticks per host but could allow for some heterogeneity.

## Conclusions

We present a package for researchers to write, run, and analyze tick population and infection dynamics models. This package will make it easier for those models to be shared, replicated, and modified since necessary configuration information is validated and stored in a single R object. Although the package structure limits the types of models it can accommodate, we think it can still be used to help researchers address critical issues in the biology, prediction, and control of ticks and tick-borne diseases in a reproducible manner. As that happens, and we hear from users what additional features or improvements are desired, we will release new versions of the package with those incorporated.

### Supplementary Information

**Additional file 1.** Appendices A–C provide examples of how to use the package to write, run, and then analyze tick population models. A) replicates the Ixodes scapularis model from Ogden et al. [16], B) gives a novel Dermacentor albipictus population model, and C) proviodes an example of including a tick-borne pathogen.

## Data Availability

The R package IxPopDyMod is available on the Comprehensive R Archive Network (https://cran.r-project.org/web/packages/IxPopDyMod/index.html). The appendices provide the R code necessary to reproduce the results contained. Other examples of package use can be found in the package readme at https://github.com/dallenmidd/IxPopDyMod#readme.
