# Supplementary material for: IxPopDyMod: an R package to write, run, and analyze tick population and infection dynamics models
Source: Parasit Vectors. 2024 Feb 26;17:90. doi: 10.1186/s13071-024-06171-2 (PMC10898031; doi:10.1186/s13071-024-06171-2)
Supplement: Supplementary file 1 — Additional file 1. Appendices A–C provide examples of how to use the package to write, run, and then analyze tick population models. A) replicates the Ixodes scapularis model from Ogden et al. [16], B) gives a novel Dermacentor albipictus population model, and C) proviodes an example of including a tick-borne pathogen. [file 13071_2024_6171_MOESM1_ESM.pdf]

Appendix A Replicating Ogden et al. (2005)'s model

Ogden et al. [1] give a population dynamics model for *Ixodes scapularis*, the black-legged tick, which is the main vector of the Lyme disease agent in eastern North America. Here we demonstrate that we can largely replicate this model in our package framework. We include the model in our package with the `ogden2005` config. To help users with of the package, in this appendix we provide all of the R code necessary to specify the model configuration, run it, and produce graphs to replicate those in [1].

Differences with Ogden et al. [1]

We were able to largely replicate Ogden et al.'s [1] model directly with our package, but make a few changes:

- The model in [1] has density-dependent reduction in fecundity based on the number of adult ticks on deer. We take fecundity (number of eggs produced) as a constant.
- The model in [1] handles host finding differently:
  - They give a weekly probability of host-finding. We translate this into a daily probability.
  - They use curves (Figure 3 from [1]) for life-stage specific probabilities that a tick quests based on temperature. We approximate these curves with Briere functions.
- To get the temperature inputs for the default model run we digitized the data from Figure 2 in [1].
- For the model runs under different climates (Figure 7 in [1]) we downloaded temperature inputs from [2].

Even with these slight differences we are able to qualitatively replicate the model's results in [1].

Specifying the model

Here is the R code to specify the Ogden et al. [1] model in our package. We give life stages a three character name. The first character is the process that life stage is going through: `h` for hardening, `q` for questing (host seeking), `a` for attached to a host, `e` for engorged, and `r` for reproductive. The second character is for infected or uninfected, this model does not include a tick-borne pathogen so this is left blank, `_`. The last character gives the broad life stage: `e` for egg, `l` for larva, `n` for nymph, and `a` for adult. So, for example, `q_n` is a questing nymph.

```
library(IxPopDyMod)
library(tidyverse)
ogden2005 <- config(
  life_cycle(
    transition("__e", "h_l", expo_fun, "duration",
               predictors = list(x = predictor_spec("temp", FALSE)),
               parameters = list(a = 2.92e-05, b = 2.27)),
    transition("__e", NULL, constant_fun, "duration",
```

```
             mortality_type = "per_day",
             parameters = list(a = 0.002)),
  transition("e_l", NULL, constant_fun, "duration",
             mortality_type = "per_day",
             parameters = list(a = 0.003)),
  transition("e_l", "q_n", expo_fun, "duration",
             predictors = list(x = predictor_spec("temp", FALSE)),
             parameters = list(a = 9.883278e-06, b = 2.55)),
  transition("e_n", NULL, constant_fun, "duration",
             mortality_type = "per_day",
             parameters = list(a = 0.002)),
  transition("e_n", "q_a", expo_fun, "duration",
             predictors = list(x = predictor_spec("temp", FALSE)),
             parameters = list(a = 0.0006265664, b = 1.21)),
  transition("a_l", "e_l", constant_fun, "duration",
             parameters = list(a = 0.5)),
  transition("a_n", "e_n", constant_fun, "duration",
             parameters = list(a = 0.25)),
  transition("h_l", "q_l", constant_fun, "duration",
             parameters = list(a = 0.0476)),
  transition("h_l", NULL, constant_fun, "duration",
             mortality_type = "per_day",
             parameters = list(a = 0.006)),
  transition("q_l", NULL, constant_fun, "probability",
             mortality_type = "per_day",
             parameters = list(a = 0.006)),
  transition("e_a", "r_a", expo_fun, "duration",
             predictors = list(x = predictor_spec("temp", FALSE)),
             parameters = list(a = 0.0007692308, b = 1.42)),
  transition("r_a", "__e", constant_fun, "probability",
             parameters = list(a = 3000)),
  transition("e_a", NULL, constant_fun, "duration",
             mortality_type = "per_day",
             parameters = list(a = 1e-04)),
  transition("a_a", "e_a", constant_fun, "duration",
             parameters = list(a = 0.111)),
  transition("q_a", NULL, constant_fun, "probability",
             mortality_type = "per_day",
             parameters = list(a = 0.006)),
  transition("q_n", NULL, constant_fun, "probability",
             mortality_type = "per_day",
             parameters = list(a = 0.006)),
  transition("q_l", "a_l", ogden_feed_fun, "probability",
             predictors = list(x = predictor_spec("temp")),
             parameters = list(a = 0.0207, q = 7e-04,
```

```
                                   tmax = 35, tmin = 10)),
    transition("q_n", "a_n", ogden_feed_fun, "probability",
               predictors = list(x = predictor_spec("temp")),
               parameters = list(a = 0.0207, q = 7e-04,
                                   tmax = 35, tmin = 10)),
    transition("q_a", "a_a", ogden_feed_fun, "probability",
               predictors = list(x = predictor_spec("temp")),
               parameters = list(a = 0.0459, q = 0.0088,
                                   tmax = 16, tmin = 3)),
    transition("a_l", NULL, density_fun, "duration",
               mortality_type = "throughout_transition",
               predictors = list(x = predictor_spec("host_den"),
                                   y = predictor_spec("[af].."),
               parameters = list(a = 0.65, b = 0.049, c = 1.01,
                                   pref = c(deer = 0, mouse = 1))),
    transition("a_n", NULL, density_fun, "duration",
               mortality_type = "throughout_transition",
               predictors = list(x = predictor_spec("host_den"),
                                   y = predictor_spec("[af].."),
               parameters = list(a = 0.55, b = 0.049, c = 1.01,
                                   pref = c(deer = 0, mouse = 1))),
    transition("a_a", NULL, density_fun, "duration",
               mortality_type = "throughout_transition",
               predictors = list(x = predictor_spec("host_den"),
                                   y = predictor_spec("[af].."),
               parameters = list(a = 0.5, b = 0.049, c = 1.01,
                                   pref = c(deer = 1, mouse = 0)))
  ),
  initial_population = c(q_a = 10000),
  steps = 3500,
  preds = readr::read_csv("predictors.csv")
)
```

Understanding `transition` objects

Here we explain two `transition` objects to show how they work. The first transition gives development from eggs (`__e`) to hardening larvae (`h_l`). This is a duration based transition and the daily rate it happens is an exponential function of temperature. Each day the fraction of development that takes place is $2.92 \times 10^{-05} \times \text{temp}^{2.27}$.

The final transition gives the mortality of adult ticks attached to hosts (`a_a`). Because of their preference they are only attached to deer. This mortality is given as the total mortality attached adults experience not their daily mortality (because `mortality_type` is `throughout_transition` rather than `per_day`). This mortality rate is density dependent based on the number of attached and feeding adults on deer. The mortality rate is $0.5 + \frac{0.049 \times \ln(1.01 + \text{FA})}{D}$ where FA is the number of feeding and

attached adults and D is the number of deer. This function, as with all transition functions, is taken from [1].

Figure 4 from [1]

Figure 4 in [1] shows that their model can replicate questing tick seasonality patterns from Long Point, Ontario, Canada. Here we show that our version largely follows their model output. For this graph we digitized the model predicted values from Figure 4 in [1]. We omit the observed values here, because our purpose is to show we can replicate [1]'s model output rather than whether we are replicating observed data.

```
our_mod <- run(ogden2005)
ogd_mod <- read.csv('fig4_data/fig4_pred_data.csv')

# take just actively questing ticks 6 yrs into the run get stabilized population
questing <- our_mod %>%
  mutate(post_eq_jday = day - 365 * 6) %>%
  filter(post_eq_jday > 0,
         post_eq_jday < 365 * 2,
         str_detect(stage,'a_'))

# get julian days shown in Fig 4 for each life stage
fig4_jdays_l <- ogd_mod %>%
  filter(stage == 'pl') %>%
  pull(jday) %>%
  unique()
fig4_jdays_n <- ogd_mod %>%
  filter(stage == 'pn') %>%
  pull(jday) %>%
  unique()
fig4_jdays_a <- ogd_mod %>%
  filter(stage == 'pa') %>%
  pull(jday) %>%
  unique()


# prepare our data for merge
our_to_merge <- questing %>%
  filter((post_eq_jday %in% fig4_jdays_l & stage == 'a_l') |
         (post_eq_jday %in% fig4_jdays_n & stage == 'a_n') |
         (post_eq_jday %in% fig4_jdays_a & stage == 'a_a') ) %>%
  select(jday = post_eq_jday,
            num = pop,
            stage = stage
           )
```

```
merged_fig4 <- rbind(ogd_mod, our_to_merge) %>%
  mutate(which_model = ifelse(str_detect(stage,'a_'),'IxPopDyMod','Ogden 2005'),
         life_stage = case_match(stage,
                                 c('pa', 'a_a') ~ 'Adult',
                                 c('pn', 'a_n') ~ 'Nymph',
                                 c('pl', 'a_l') ~ 'Larva')) %>%
  # scale both to proportion of total questing that year (y axis in Figure 4)
  group_by(which_model, life_stage) %>%
  mutate(num = num/sum(num)) %>%
  ungroup() %>%
  mutate(life_stage = factor(life_stage, levels = c('Larva', 'Nymph', 'Adult')))

merged_fig4 %>%
  ggplot(aes(jday, num, shape = which_model, linetype = which_model)) +
  geom_point() +
  geom_line() +
  theme_classic() +
  xlab('Day of year') +
  ylab('Proportion of year total') +
  scale_linetype_discrete(name = "Which model") +
  scale_shape_discrete(name = "Which model") +
  facet_wrap(~life_stage, dir = 'v')
```

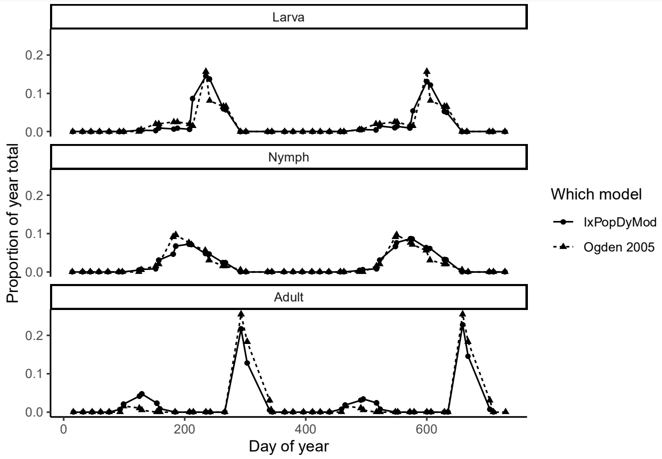

This figure shows the predicted blacklegged tick host-seeking seasonality and how it differs by tick life stage. As expected for blacklegged ticks in northeastern North America: larvae are active in late summer/early fall (peaking around Julian day 250, September 7); nymphs are active earlier in the summer (peaking around Julian day 190, July 9); and adults are active in both early spring and late fall.

Figure 7 from [1]

Figure 7 in [1] shows the predicted number of ticks at a range of locations in Canada. Here we reproduce the figure for a subset of locations, just six from across the latitudinal range in Ontario. We are looking to replicate the pattern seen in Figure 7 for these sites. Figure 7 shows that tick density is near zero until degree days reach about 2750 C and then increases linearly from there. In other words the blacklegged tick population is highly sensitive to total thermal accumulation (as measured by degree days), with near zero populations when the degree days is below 2750 C.

```
# function to quickly change the weather in an existing model config
set_weather <- function(cfg, weather) {
  temp_weather <- read_csv(paste0(
    "fig7_data/weather_",
    weather, ".csv"
  ))
  cfg$preds <- tibble(
    value = c(20, 200, temp_weather$tmean),
    j_day = c(NA, NA, temp_weather$j_day),
    pred = c("host_den", "host_den", rep("temp", dim(temp_weather)[1])),
    pred_subcategory = c("deer", "rodent", rep(NA, dim(temp_weather)[1]))
  )

  return(cfg)
}

locations <- c(
  "exeter", "hanover", "kapuskasing_cda",
  "new_glasgow", "point_pelee", "south_baymouth"
)

configs <- sapply(locations, function(x) {
  set_weather(ogden2005, x)
},
simplify = FALSE
)

outputs <- lapply(configs, run)

dfs <- lapply(outputs, function(df) filter(df, stage == 'a_a'))

# return the max number of adults during the ninth full calendar year
# (which is the last full year if model was run for 3500 steps)
max_adults_ninth_year <- function(out_N_df) {
  out_N_df %>%
    filter(day > 365 * 8, day < 365 * 9) %>%
    pull(pop) %>%
```

```r
    max()
}

mean_dd_gt_zero <- c(3336, 3100, 2317, 3536, 3791, 2733)

fig7_tbl <- tibble(
  dd = mean_dd_gt_zero,
  max_adults = unlist(lapply(dfs, max_adults_ninth_year))
)

ggplot(fig7_tbl, aes(mean_dd_gt_zero, max_adults)) +
  geom_point() +
  xlim(2000, 4000) +
  xlab("Mean annual degree-days >0 °C") +
  ylab("Maximum no. adult ticks at equilibrium") +
  theme_classic()
```

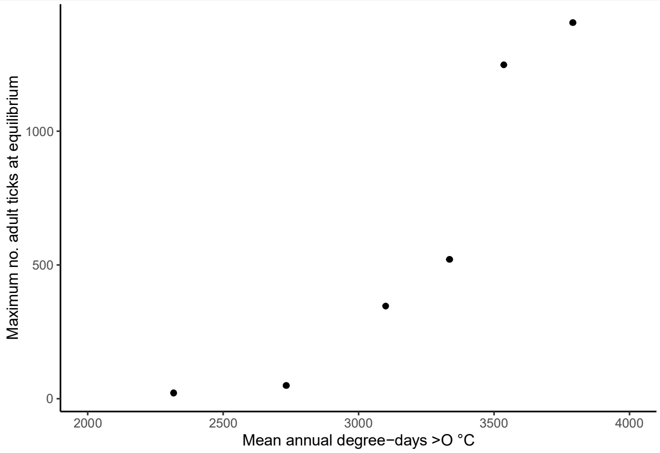

Our model does reproduce the pattern seen in [1]'s model.

References

1. Ogden NH, Bigras-Poulin M, O'Callaghan CJ, Barker IK, Lindsay LR, Maarouf A, Smoyer-Tomic KE, Waltner-Toews D, Charron D. A dynamic population model to investigate effects of climate on geographic range and seasonality of the tick *Ixodes scapularis*. Int J Parasitol. 2010;35: 375-389.
2. Government of Canada. Canadian Climate Normals. https://climate.weather.gc.ca/climate_normals/index_e.html. Accessed 10 May 2023.

Appendix B Winter tick population model

In this appendix, we use IxPopDyMod to specify a model that reproduces the *Dermacentor albipictus* winter tick biology observed in Alberta, Canada from Drew and Samuel [1].

Assembling predictor data to use in model

For the *D. albipictus* population model we use three predictors informed by the biology of this tick: temperature, snow cover, and moose population density. Temperature affects the rate at which larvae quest for a host and off-host development rate from engorged to reproductive adults. Snow cover affects off-host tick survival. Moose population density affects the rate at which larval ticks find a host.

Drew and Samuel [1] present weekly maximum temperatures, but IxPopDyMod expects daily weather data. We get this from a weather station at Elk Island (near the study site), whose data is accessible via the Canadian Government's historical climate data portal [2]. Here we show these data are similar to the weekly data from [1].

```
library(dplyr)
library(readr)
library(ggplot2)

# Digitized temperature data from Drew and Samuel
weekly_max_temp <- read_csv("drew_samuel_1986_fig5_grassland_digitized.csv")

# weather data from [2]
daily_weather <- read_csv("en_climate_daily_AB_3012275_1982_P1D.csv")

comparison <- daily_weather %>%
  mutate(
    j_day = as.POSIXlt(`Date/Time`)$yday,
    data_source = "Weather station"
  ) %>%
  select(j_day, data_source, temp = `Max Temp (°C)`) %>%
  bind_rows(weekly_max_temp %>%
              mutate(data_source = "Drew and Samuel (1986)"))

comparison %>%
  ggplot(aes(x = j_day, y = temp, color = data_source)) +
  geom_line() +
  geom_point() +
  xlab("Julian day") +
  ylab("Temperature (ºC)") +
  labs(color = "Data source")
```

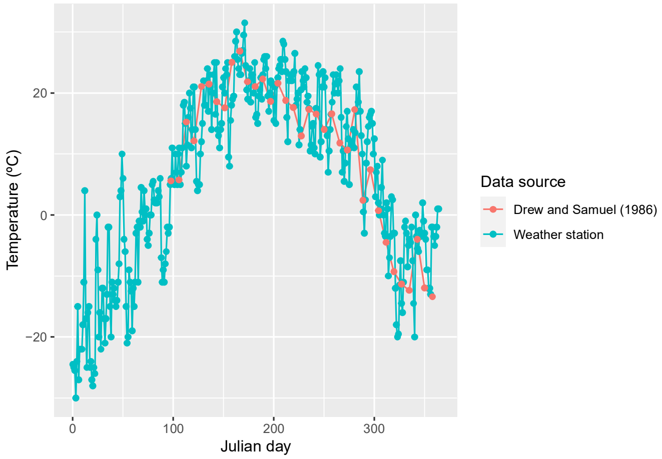

As these are similar, we use the weather station data to reproduce the field study's results with our model. We now reshape the weather data into a `IxPopDyMod::predictors()` object to use it in the model. For details on the required structure, see the `IxPopDyMod::predictors()` documentation. In addition to temperature, we include snow cover in the predictors since it affects questing larva and engorged adult mortality.

```
library(tidyr)
predictors <- daily_weather %>%
  mutate(j_day = 1:365) %>%
  rename(max_temp = `Max Temp (°C)`,
         snow_cover = `Snow on Grnd (cm)`) %>%
  pivot_longer(
    cols = c(max_temp, snow_cover),
    values_to = "value", names_to = "pred"
  ) %>%
  mutate(
    pred_subcategory = NA,
    value = ifelse(is.na(value), 0, value)
  ) %>%
  select(pred, pred_subcategory, j_day, value)
```

To model population over multiple years, we repeat the weather data ten times.

```
accumulate <- predictors
for (i in 1:10) {
  accumulate <- bind_rows(accumulate, predictors)
}
days <- seq_len(nrow(accumulate) / 2)
days <- c(days, days) %>% sort()
accumulate$j_day <- days
```

```
offset <- 155
accumulate <- accumulate %>%
  mutate(true_j_day = j_day) %>%
  filter(true_j_day > offset) %>%
  mutate(j_day = j_day - offset)
```

Finally, we add host density data to the predictors table. The only host we're concerned with in this case is moose (*Alces americanus*). The `j_day` is set as `NA` to indicate that this is a constant predictor, i.e. it does not change over time.

```
# add a row for host density
accumulate <- bind_rows(
  data.frame(pred="host_den",
             pred_subcategory="moose",
             j_day=NA,
             value=0.18),
  accumulate
)
head(accumulate)
```

```
##         pred pred_subcategory j_day value true_j_day
## 1   host_den            moose    NA  0.18         NA
## 2   max_temp             <NA>     1  9.50        156
## 3 snow_cover             <NA>     1  0.00        156
## 4   max_temp             <NA>     2  8.00        157
## 5 snow_cover             <NA>     2  0.00        157
## 6   max_temp             <NA>     3 15.50        158
```

Configuring the model

We created this *D. albipictus* population model by drawing from literature on how temperature, snow cover, and host population density affect its life history processes [1,3-5]. For parameters not well described or understood in the literature, we used the model to deduce reasonable parameter values. The model configuration is as follows. We abbreviate life stages with the same convention as Appendix A.

```
library(IxPopDyMod)

winter_tick <- config(
  life_cycle(
    transition(
      "__e",
      "q_l",
      constant_fun,
      "duration",
      parameters = list(a = 0.0125)),
    transition(
      "__e",
```

```
    NULL,
    constant_fun,
    "duration",
    mortality_type = 'throughout_transition',
    parameters = list(a = 0.5)
  ),
  transition(
    "q_l",
    "a_l",
    feed_fun,
    "probability",
    predictors = list(
      x = predictor_spec('host_den'),
      y = predictor_spec('max_temp')),
    parameters = list(
      a = 0.1,
      pref = 1,
      q = 7e-4,
      tmax = 35,
      tmin = 0
    )
  ),
  transition(
    'q_l',
    NULL,
    snow_cover_fun,
    "probability",
    mortality_type = 'per_day',
    predictors = list(x = predictor_spec('snow_cover')),
    parameters = c(no_snow_mort = 0.06, snow_mort = 0.95)
  ),
  transition(
    'a_l',
    'e_a',
    constant_fun,
    'duration',
    parameters = list(a = 0.00526)),
  transition(
    'a_l',
    NULL,
    constant_fun,
    'duration',
    mortality_type = 'throughout_transition',
    parameters = list(a = 0.97)
  ),
```

```
    transition(
      'e_a',
      'r_a',
      expo_fun,
      'duration',
      predictors = list(
        x = predictor_spec(
          'max_temp',
          first_day_only = FALSE)),
      parameters = list(a = 0.0002, b = 1.67)
    ),
    transition(
      'e_a',
      NULL,
      snow_cover_fun,
      'duration',
      predictors = list(x = predictor_spec('snow_cover')),
      mortality_type = 'throughout_transition',
      parameters = c(no_snow_mort = 0.36, snow_mort = 0.89)
    ),
    transition(
      'r_a',
      '__e',
      constant_fun,
      'probability',
      parameters = c(a = 3000))
  ),
  initial_population = c(r_a = 10),
  steps = 1500,
  preds = accumulate
)
```

Run the model

Now we run the model and calculate the annual growth rate.

```
model_results <- run(winter_tick, progress = FALSE)
annual_growth_rate(model_results)
```

```
## [1] 0.9187258
```

Since the annual growth rate is less than 1 the population is decreasing. It changes by a factor of 0.92 every year, so in other words decreases by 8% per year. Here is how the number of ticks of each life stage changes over the first two years of the model run.

```
model_results %>%
  filter(day < 365*2) %>%
  mutate(stage = factor(stage,
```

```
                        levels = c('__e', 'q_l', 'a_l', 'e_a', 'r_a'))) %>%
  ggplot(aes(x = day, y = pop, color = stage)) +
    geom_line() +
    scale_y_log10(limits = c(1, NA)) +
    xlab("Day") +
    ylab("Population") +
    labs(col = "Tick life stage")
```

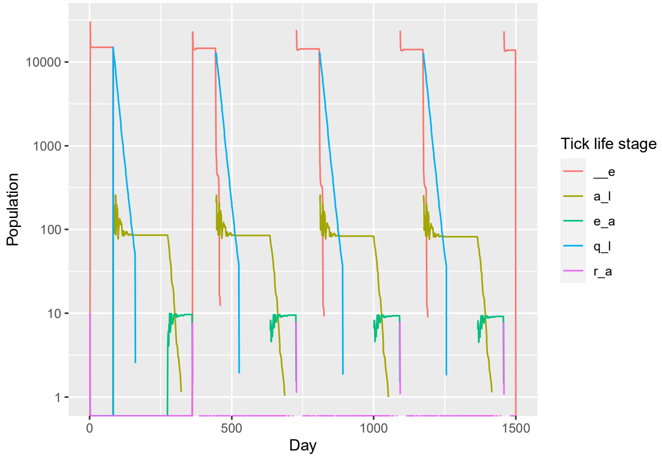

Eggs develop over the start of the run (flat red line) and drop to zero when eggs hatch and become questing larvae. The number of questing larvae drops steadily as they either die or find a host. Attached larvae stay roughly constant as they feed and develop on the moose until they are engorged adults. These then develop into reproductive adults, which die and lay eggs.

Modifying moose density

Now that we have a fairly stable model output, we can modify it, for example to see the impacts of different host community or climate dynamics on the tick population.

First, we re-run the model with a range of moose densities.

```
densities <- 0.18 * c(0.1, 0.5, 1, 2, 10)

configs <- lapply(
  densities,
  function(value) {
    cfg <- winter_tick
    cfg$preds[1, "value"] <- value
    return(cfg)
  }
)

results <- lapply(configs, run, progress = FALSE)
```

To compare the results, we plot the total tick population over time for each moose density.

```
# We add a column to each dataframe to identify what parameterization it was
# created with
for (i in seq_along(results)) {
  results[[i]]$density <- as.factor(densities[[i]])
}

# Then we combine all the results, to facilitate plotting them together
all_results <- bind_rows(results)

all_results %>%
  group_by(density, day) %>%
  summarize(total_population = sum(pop), .groups = "drop") %>%
  ggplot(aes(x = day, y = total_population, col = density)) +
  geom_line() +
  scale_y_log10(limits = c(1, NA)) +
  labs(col = "Moose per km^2") +
  xlab("Day") +
  ylab("Total tick population")
```

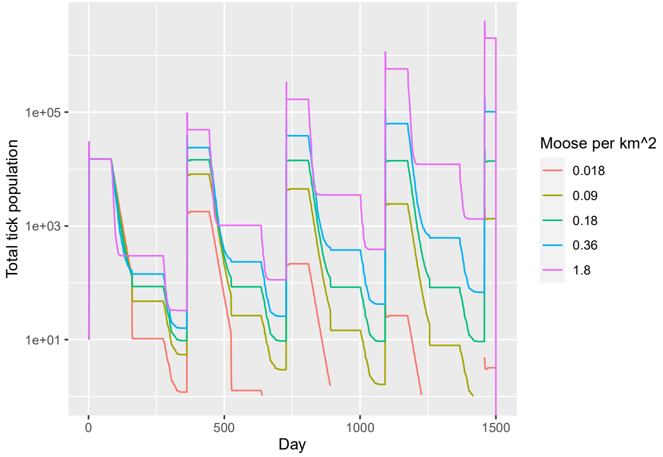

As expected, modeled tick populations are larger with higher moose density. We can also see this by calculating the annual growth rate under each scenario:

```
growth_rates <- lapply(results, annual_growth_rate)
names(growth_rates) <- paste("Growth rate with moose density =", densities)
growth_rates
```

```
## $`Growth rate with moose density = 0.018`
## [1] 0.09589349
##
```

```
## $`Growth rate with moose density = 0.09`
## [1] 0.4346676
##
## $`Growth rate with moose density = 0.18`
## [1] 0.9187258
##
## $`Growth rate with moose density = 0.36`
## [1] 1.65519
##
## $`Growth rate with moose density = 1.8`
## [1] 3.446101
```

Tick population change is highly dependent on the moose density. At the lowest density (0.018 per km2) the tick population changes by a factor of 0.1, so decreases 90% per year. While at the highest (1.8 km2) it increases by a factor of 3.4, more than triples every year.

Modifying climate

Here, we run the model under three climate scenarios. We compare the baseline (used above) to a cold scenario where the temperature is decreased by 2.5 C on every day and a warm scenario where the temperature is elevated by 2.5 C on every day. These commands copy the `winter_tick config` into two new `configs` and modify the temperature `pred` in them.

```
winter_tick_warm <- winter_tick
winter_tick_warm$preds <-
  winter_tick_warm$preds %>%
  mutate(value = ifelse(pred == "max_temp", value + 2.5, value))

winter_tick_cold <- winter_tick
winter_tick_cold$preds <-
  winter_tick_cold$preds %>%
  mutate(value = ifelse(pred == "max_temp", value - 2.5, value))
```

Now we run the different climate scenarios and compare the results.

```
warm_run <- run(winter_tick_warm, progress = FALSE)
cold_run <- run(winter_tick_cold, progress = FALSE)

cold_run$temp <- "Cold"
warm_run$temp <- "Warm"
model_results$temp <- "Baseline"

all_temp_results <- bind_rows(cold_run, warm_run, model_results)
all_temp_results %>%
  group_by(temp, day) %>%
  summarize(pop = sum(pop), .groups = "drop") %>%
  ggplot(aes(x = day,
```

```
              y = pop,
              col = factor(temp, levels = c("Warm", "Baseline", "Cold")))) +
  geom_line() +
  scale_y_log10(limits = c(1, NA)) +
  scale_color_manual(values = c(Cold = "#2c7bb6",
                                Warm = "#d7191c",
                                Baseline = "#757473")) +
  xlab("Day") +
  ylab("Total tick population") +
  labs(col = "Temperature")
```

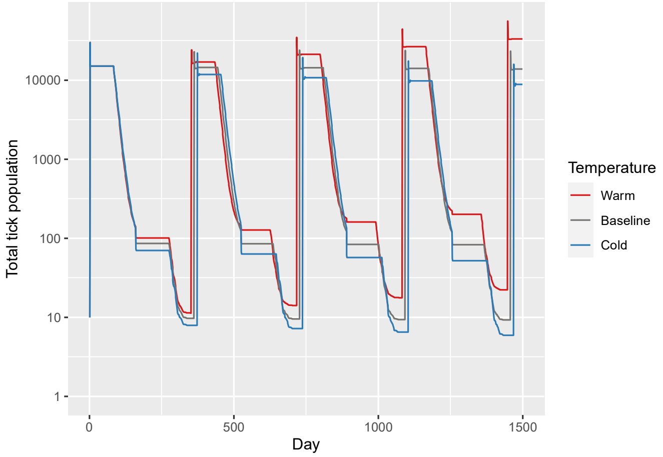

The phenology and populations are both affected by temperature. Eggs are laid (the only times that the population increases) earlier in the year under the warm climate scenario. Additionally, total population is larger under the warmer climate scenarios. This is also reflected in the average annual growth rates.

```
annual_growth_rate(cold_run)
```

```
## [1] 0.8348823
```

```
annual_growth_rate(model_results)
```

```
## [1] 0.9187258
```

```
annual_growth_rate(warm_run)
```

```
## [1] 1.229096
```

References

1. Drew ML, Samuel WM. Reproduction of the winter tick, *Dermacentor albipicuts*, under field conditions in Alberta, Canada. Can J Zool. 1986;64: 714-721.
2. Government of Canada. Historical Data. https://climate.weather.gc.ca/historical_data/search_historic_data_e.html. Accessed 10 May 2023.

3. Addison EM, McLaughlin RF. Growth and development of winter tick, *Dermacentor albipicuts*, on moose, *Alces alces*. J Parasitol. 1988;74: 670-678.
4. Drew ML, Samuel WM. Factors affecting transmission of larval winter ticks, *Dermacentor albipicuts* (Packard), to moose, *Alces alces* L., in Alberta, Canada. J Wildlife Dis. 1985;21: 274-282.
5. Samuel WM, Welch DA. Winter ticks on moose and other ungulates: factors influencing their population size. Alces. 1991;27: 168-182.

Appendix C Example with a tick-borne pathogen

Here we use `IxPopDyMod` to model both tick population and infection dynamics. We consider a population of blacklegged ticks (*Ixodes scapularis*) and the tick-borne pathogen *Borrelia burgdorferi*, the Lyme disease agent. We take a simplified two-host model with deer which feed all three tick life stages and mice which feed larvae and nymphs. To show a possible application, we look at the question of whether white-tailed deer (*Odocoileus virginianus*) can function as dilution hosts [1,2]. Deer are generally considered only as reproductive hosts of adult ticks, but they can also feed juvenile ticks. Since deer have very low reservoir competence [3], juvenile ticks which feed on deer — rather than mice — are less likely to be infected. So as deer density increases tick density may increase but the rate of infection could decrease. It is unclear if the overall effect is an increase or decrease in the density of infected ticks. We briefly illustrate how a model could address this question.

The `config` for this model

First we define a custom function to be used in the model. This function takes host finding as a Bernoulli trial. If there are x hosts and the chance of finding any one host is a, then the probability of a tick finding a host is $1-(1-a)^x$. Here $(1-a)$ is the probability of not finding any one particular host, so $(1-a)^x$ is the probability of not finding any host at all. One minus this value is the probability of finding at least one host. The $pref$ term allows for preferences between different host species.

```
find_host <- function(x, a, pref) {
  1 - (1 - a)^sum(x * pref)
}
```

With this custom function defined we can specify the full model:

```
library(IxPopDyMod)
infect_example_config <- config(
  life_cycle(
    transition("__e", "q_l", constant_fun, "duration",
               parameters = list(a = 0.02)),
    transition("__e", NULL, constant_fun, "duration",
               mortality_type = 'per_day',
               parameters = list(a = 0.01)),
    transition("q_l", "f_l", find_host, "probability",
               predictors = list(x = predictor_spec("host_den")),
               parameters = list(a = 1e-4, pref = c(deer = 1, mouse = 0.05))),
    transition("q_l", NULL, constant_fun, "probability",
               mortality_type = 'per_day', parameters = list(a = 0.01)),
    transition("f_l", "eil", infect_fun, "probability",
               predictors = list(x = predictor_spec("host_den")),
               parameters = list(from_infected = 0, to_infected = 1,
                                 host_rc = c(deer = 0.01, mouse = 0.5),
                                 pref = c(deer = 1, mouse = 0.05))),
```

```
    transition("f_l", "eul", infect_fun, "probability",
               predictors = list(x = predictor_spec("host_den")),
               parameters = list(from_infected = 0, to_infected = 0,
                                 host_rc = c(deer = 0.01, mouse = 0.5),
                                 pref = c(deer = 1, mouse = 0.05))),
    transition("eil", "qin", constant_fun, "duration",
               parameters = list(a = 0.025)),
    transition("eil", NULL, constant_fun, "duration",
               mortality_type = 'per_day',
               parameters = list(a = 0.01)),
    transition("eul", "qun", constant_fun, "duration",
               parameters = list(a = 0.025)),
    transition("eul", NULL, constant_fun, "duration",
               mortality_type = 'per_day',
               parameters = list(a = 0.01)),
    transition("qin", "fin", find_host, "probability",
               predictors = list(x = predictor_spec("host_den")),
               parameters = list(a = 1e-4, pref = c(deer = 1, mouse = 0.05))),
    transition("qin", NULL, constant_fun, "probability",
               mortality_type = 'per_day',
               parameters = list(a = 0.01)),
    transition("qun", "fun", find_host, "probability",
               predictors = list(x = predictor_spec("host_den")),
               parameters = list(a = 1e-4, pref = c(deer = 1, mouse = 0.05))),
    transition("qun", NULL, constant_fun, "probability",
               mortality_type = 'per_day',
               parameters = list(a = 0.01)),
    transition("fun", "ein", infect_fun, "probability",
               predictors = list(x = predictor_spec("host_den")),
               parameters = list(from_infected = 0, to_infected = 1,
                                 host_rc = c(deer = 0.01, mouse = 0.5),
                                 pref = c(deer = 1, mouse = 0.05))),
    transition("fun", "eun", infect_fun, "probability",
               predictors = list(x = predictor_spec("host_den")),
               parameters = list(from_infected = 0, to_infected = 0,
                                 host_rc = c(deer = 0.01, mouse = 0.5),
                                 pref = c(deer = 1, mouse = 0.05))),
    transition("fin", "ein", infect_fun, "probability",
               predictors = list(x = predictor_spec("host_den")),
               parameters = list(from_infected = 1, to_infected = 1,
                                 host_rc = c(deer = 0.01, mouse = 0.5),
                                 pref = c(deer = 1, mouse = 0.05))),
    transition("ein", "qia", constant_fun, "duration",
               parameters = list(a = 0.025)),
    transition("ein", NULL, constant_fun, "duration",
```

```
                 mortality_type = 'per_day',
                 parameters = list(a = 0.01)),
    transition("eun", "qua", constant_fun, "duration",
                 parameters = list(a = 0.025)),
    transition("eun", NULL, constant_fun, "duration",
                 mortality_type = 'per_day',
                 parameters = list(a = 0.01)),
    transition("qia", "fia", find_host, "probability",
                 predictors = list(x = predictor_spec("host_den")),
                 parameters = list(a = 1e-4, pref = c(deer = 1, mouse = 0))),
    transition("qia", NULL, constant_fun, "probability",
                 mortality_type = 'per_day',
                 parameters = list(a = 0.01)),
    transition("qua", "fua", find_host, "probability",
                 predictors = list(x = predictor_spec("host_den")),
                 parameters = list(a = 1e-4, pref = c(deer = 1, mouse = 0))),
    transition("qua", NULL, constant_fun, "probability",
                 mortality_type = 'per_day',
                 parameters = list(a = 0.01)),
    transition("fua", "eia", infect_fun, "probability",
                 predictors = list(x = predictor_spec("host_den")),
                 parameters = list(from_infected = 0, to_infected = 1,
                                   host_rc = c(deer = 0.01, mouse = 0.5),
                                   pref = c(deer = 1, mouse = 0))),
    transition("fua", "eua", infect_fun, "probability",
                 predictors = list(x = predictor_spec("host_den")),
                 parameters = list(from_infected = 0, to_infected = 0,
                                   host_rc = c(deer = 0.01, mouse = 0.5),
                                   pref = c(deer = 1, mouse = 0))),
    transition("fia", "eia", infect_fun, "probability",
                 predictors = list(x = predictor_spec("host_den")),
                 parameters = list(from_infected = 1, to_infected = 1,
                                   host_rc = c(deer = 0.01, mouse = 0.5),
                                   pref = c(deer = 1, mouse = 0))),
    transition("eia", "r_a", constant_fun, "duration",
                 parameters = list(a = 0.025)),
    transition("eia", NULL, constant_fun, "duration",
                 mortality_type = 'per_day',
                 parameters = list(a = 0.01)),
    transition("eua", "r_a", constant_fun, "duration",
                 parameters = list(a = 0.025)),
    transition("eua", NULL, constant_fun, "duration",
                 mortality_type = 'per_day',
                 parameters = list(a = 0.01)),
    transition('r_a','__e', constant_fun, 'probability',
```

```
                parameters = list(a = 500))
  ),
  initial_population = c(r_a = 10),
  steps = 500,
  preds = data.frame(
    pred = "host_den",
    pred_subcategory = c("deer", "mouse"),
    j_day = NA,
    value = c(0.5, 15)
  )
)
```

We maintain the same naming convention from the Appendices A and B, but since we have infection in this model the middle character is used. It is `i` for infected individuals, `u` for uninfected, and `_` for life stages without the possibility of infection. Here we model a tick-borne pathogen without transovarial infection so reproductive adults, eggs, and pre-feeding larvae are in this last category. The model could be modified to include transovarial infection: infected reproductive adults, `ria`, would give rise to infected eggs, `_ie`.

The key to this model is that feeding uninfected ticks can either become engorged infected or uninfected ticks. This is based on the `infect_fun` transition function, which draws on the host-species specific feeding preference and reservoir competence parameters.

What fraction of questing nymphs are infected?

Now we can run the model and see what fraction of questing nymphs are infected. These nymphs would have been infected during their first bloodmeal as larvae. This value called the nymphs infection prevalence (NIP), is of importance to public health since nymphs are the stage that most often transmit the *B. burgdorferi* pathogen to people.

```
output <- run(infect_example_config)
tot_qin <- sum(output[output$stage == 'qin','pop'])
tot_qun <- sum(output[output$stage == 'qun','pop'])

tot_qin/(tot_qin + tot_qun)
```

```
## [1] 0.304
```

The model predicts that about 30% of questing nymphs should be infected.

Effect of deer density

As deer density increases this should increase the total tick population by providing more tick hosts. At the same time it should decrease NIP, since larvae that would have fed on mice are now feeding on deer. Here we can confirm that the model reproduces these intuitive results.

```r
deer_den <- c(0.1, 0.25, 0.5, 0.75, 1)
results_df <- data.frame(deer = deer_den, nymph_den = 0, nip = 0, din = 0)

for (i in 1:5)
{
  cfg_mod <- infect_example_config
  cfg_mod$preds[1, 4] <- deer_den[i]
  out <- run(cfg_mod)

  results_df$nip[i] <- sum(out[out$stage=='qin','pop'])/(sum(out[out$stage=='qin','pop']) + su

  results_df$nymph_den[i] <- sum(out[out$stage=='qin','pop']) + sum(out[out$stage=='qun','pop']

  results_df$din[i] <- sum(out[out$stage=='qin','pop'])
}

plot(results_df$deer,results_df$nymph_den, xlab = 'Deer density', ylab = 'Number of questing ny
```

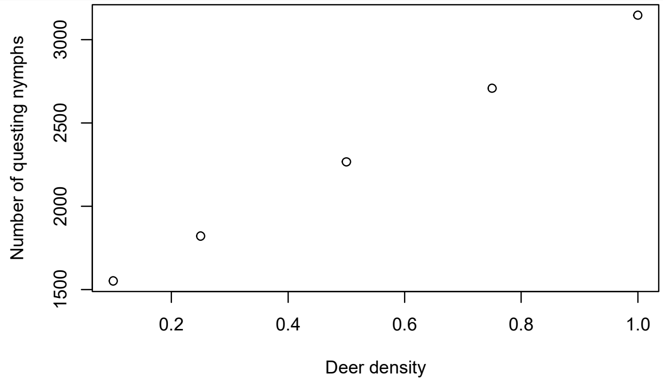


```r
plot(results_df$deer,results_df$nip, xlab = 'Deer density', ylab = 'NIP')
```

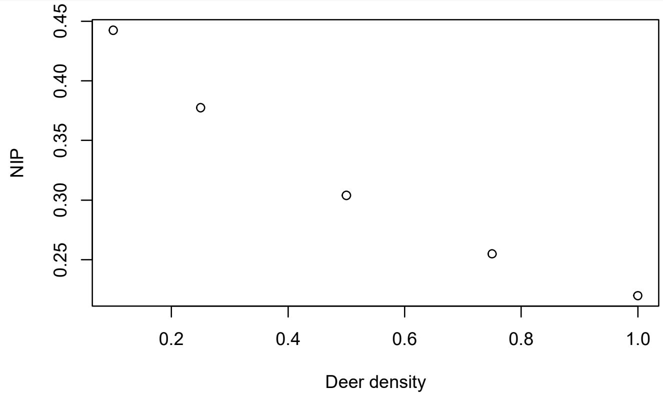

Finally the key question is whether more deer increase the density of *infected* nymphs (DIN). DIN is often thought to be a key proxy for human health risk [4].

```
plot(results_df$deer,results_df$din, xlab = 'Deer density', ylab = 'DIN')
```

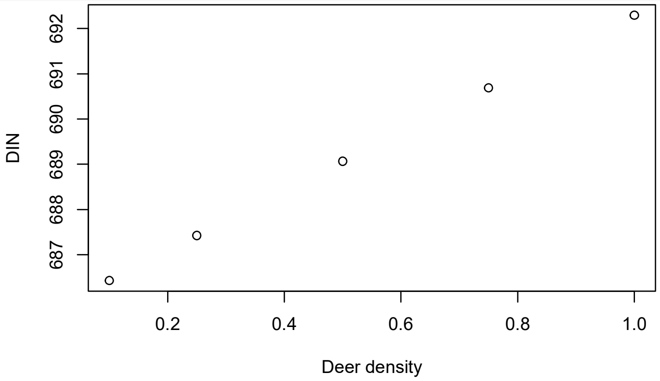

With this parametrization the model predicts a very small overall effect of deer on the density of infected nymphs (compare y axis range here to that above). The deer's decrease in NIP roughly balances their increase in nymph density to result in very little overall change in DIN. It should be emphasized that this is just a preliminary example of how the package structure could be used to look at tick-borne pathogen infection. See [1,2] for more thorough examinations of whether deer can function as dilution hosts.

References

1. Gandy S, Kilbride E, Biek R, Millins C, Gilbert L. No net effect of host density on tick-borne disease hazard due to opposing roles of vector amplification and pathogen dilution. Ecol Evol. 2022;12:e9253.
2. Huang C-I, Kay SC, Davis S, Tufts DM, Gaffett K, Tefft B, Diuk-Wasser MA. High burdens of *Ixodes scapularis* larval ticks on white-tailed deer may limit Lyme disease risk in a low biodiversity setting. Ticks Tick Borne Dis. 2019;10:258-268.
3. LoGiudice K, Ostfeld RS, Schmidt KS, Keesing F. The ecology of infectious disease: effects of host diversity and community composition on Lyme disease risk. PNAS 2003;100:567-571.
4. Eisen L, Eisen RJ. Critical evaluation of the linkage between tick-based risk measures and the occurrence of Lyme disease cases. J Med Entomol. 2016;53:1050-1062.
